# Identification of an autoantibody panel to separate lung cancer from smokers and nonsmokers

**DOI:** 10.1186/1471-2407-10-234

**Published:** 2010-05-26

**Authors:** William N Rom, Judith D Goldberg, Doreen Addrizzo-Harris, Heather N Watson, Michael Khilkin, Alissa K Greenberg, David P Naidich, Bernard Crawford, Ellen Eylers, Daorong Liu, Eng M Tan

**Affiliations:** 1 Division of Pulmonary, Critical Care, and Sleep Medicine, Department of Medicine, Radiology, Thoracic Surgery, and Environmental Medicine, New York University School of Medicine, New York, NY 10016, USA; 2 Department of Molecular and Experimental Medicine, The Scripps Research Institute, 10550 North Torrey Pines Road, La Jolla, CA, 92037, USA

## Abstract

**Background:**

Sera from lung cancer patients contain autoantibodies that react with tumor associated antigens (TAAs) that reflect genetic over-expression, mutation, or other anomalies of cell cycle, growth, signaling, and metabolism pathways.

**Methods:**

We performed immunoassays to detect autoantibodies to ten tumor associated antigens (TAAs) selected on the basis of previous studies showing that they had preferential specificity for certain cancers. Sera examined were from lung cancer patients (22); smokers with ground-glass opacities (GGOs) (46), benign solid nodules (55), or normal CTs (35); and normal non-smokers (36). Logistic regression models based on the antibody biomarker levels among the high risk and lung cancer groups were developed to identify the combinations of biomarkers that predict lung cancer in these cohorts.

**Results:**

Statistically significant differences in the distributions of each of the biomarkers were identified among all five groups. Using Receiver Operating Characteristic (ROC) curves based on age, c-myc, Cyclin A, Cyclin B1, Cyclin D1, CDK2, and survivin, we obtained a sensitivity = 81% and specificity = 97% for the classification of cancer vs smokers(no nodules, solid nodules, or GGO) and correctly predicted 31/36 healthy controls as noncancer.

**Conclusion:**

A pattern of autoantibody reactivity to TAAs may distinguish patients with lung cancer versus smokers with normal CTs, stable solid nodules, ground glass opacities, or normal healthy never smokers.

## Background

Early detection of lung cancer is critical for impacting the poor 5-year survival of 15 percent that has persisted for decades. If the cancer is detected in Stage I, survival can exceed 80 percent [1]. CT scans of the chest have greater sensitivity compared to posterior-anterior chest x-rays in detecting small non-calcified nodules that may represent early lung cancers; however, this technique has poor specificity because of the high prevalence of non-calcified and ground glass pulmonary nodules [2,3]. These nodules may be due to granulomatous disease, fibrosis, atypical adenomatous hyperplasia (AAH), bronchoalveolar carcinoma (BAC), adenocarcinoma, or slowly resolving inflammatory lesions. Ground glass opacities are hazy nodules without obscuration of underlying vascular markings. The National Lung Cancer Screening Trial has been undertaken by the National Institutes of Health to test the hypothesis that CT-scan screening reduces mortality by increased detection of Stage I and II lung cancers.

Blood tests such as serum autoantibodies may identify individuals with early lung cancer and distinguish high-risk smokers with benign noncalcified lesions from those with lung cancer. Malignant cells can activate both the cellular and humoral immune systems, leading to autoimmunity to autologous cellular antigens. Autoantibodies to p53, a tumor suppressor protein mutated in 70% of smokers' lung cancer, were detected in the serum of patients with breast cancer in 1982 [4]. The NHLBI Lung Health Study had 23 cancers in the 5-year trial and 5 (23%) had p53 autoantibodies including sera from 2 lung cancers drawn 6 and 7 months prior to diagnosis [5]. In 133 lung cancer patients, antibodies to p53 were detected in 25 (18.8%), with significant associations with squamous cell type, smoking, and advanced stage [6], and a similar study found p53 autoantibodies in 17.9% of heavy smokers with lung cancer [7]. Five percent of NSCLC sera contained antibodies to c-myc, p53, and eukaryotic translation initiation factor 4 G (eIF4G) [8]. Antibodies with specificity for antigens initially recognized by cytotoxic T lymphocytes, e.g. MAGE, tyrosinase, and NY-ESO-1 have been found in few lung cancer sera [9]. Other immunogenic tumor-associated antigens (TAAs) have been identified through the use of autoantibodies in cancer sera to immunoscreen cDNA expression libraries in order to identify cDNA clones encoding those TAAs [10-12]. Most of these TAAs are involved in cellular functions, including DNA replication and transcription, and pre-messenger RNA splicing and translation [12]. TAAs are often membrane receptors such as HER-2/neu oncoprotein, tumor suppressor gene proteins, cell cycle associated proteins (cyclin B1), centromere protein F (CENP-F), or onconeural antigens [13,14]. Autoantibodies to some of these antigens have been found in patients with breast, hepatocellular, ovarian, neuroendocrine, colorectal and lung cancers [4,15-19]. A proteomic approach using solubilized proteins from lung adenocarcinoma cell line A549 and lung tumors were subjected to two-dimensional PAGE followed by Western blot analysis in which individual sera were tested for antibodies [20]. Glycosylated Annexin I was detected in 12/30 (40%) of lung adenocarcinomas and 3/18 squamous cell carcinomas but none in 51 healthy subjects; Annexin II was similar but was more specific for lung cancer. Annexin I is expressed in bronchial epithelium and Annexin II is found in type I and II alveolar epithelial cells. Another autoantibody referred to as PGP 9.5, an ubiquitin COOH-terminal hydrolase, was also detected in 9/64 lung cancer patients and as an antigen in 2 more [21]. Chapman and colleagues recently showed that screening for antibodies in lung cancer using a panel of seven TAAs, p53, c-myc, HER2, NY-ESO-1, CAGE, MUC1, and GBU4-5, resulted in a sensitivity of 76% and specificity of 92% [22].

We hypothesized that cumulative reactivity to a selected panel of TAAs would distinguish individuals with lung cancer from those with benign pulmonary nodules, and ground glass opacities caused by inflammation, fibrosisor neoplasia [23]. Using a mini-array of TAAs (c-myc, p53, cyclin B1, p62, Koc, IMP1 and survivin), we previously showed a greatly increased frequency of positive immune reactivity in breast, lung, prostate, gastric, colorectal and hepatocellular carcinoma [23-26]. Our goal was to develop a panel of TAAs to guide CT scan screening of high risk smokers who have noncalcified nodules to determine whether they are at risk for lung cancer.

## Methods

### Study population

One hundred and fifty eight high risk tobacco smokers and asbestos-exposed individuals in the New York University Lung Cancer Biomarker Center (NYU LCBC) were evaluated with CT-scans, questionnaires, blood and pulmonary function tests at the time of entry into the cohort. They were recruited from a public utility union where they had asbestos exposure in power plants, and faculty referrals for smokers with age >50 years and >20 pack-years of smoking. Based on these CT scans, the NYU participants were classified into the following groups: no nodules (n = 35), solid nodules (n = 55), and ground glass opacities (GGO, n = 46). An additional group of 22 lung cancer cases were identified upon referral through a thoracic surgeon; phlebotomy was performed on the day of surgery and stage and histology performed during the hospitalization. A fifth group of 36 healthy non-smokers were enrolled at the Scripps General Clinical Research Center. The Scripps group was used as normal controls in this study and came from a pool of blood donors who were employees of the Scripps Medical Institutions in La Jolla, California. They were identified to be HIV-negative and to have no evidence of HBV or HCV infection. The median age and gender are shown in Tables 1 and 2. This group of normal, non-smoking controls are a portion of a larger pool of donors available to investigators with IRB approval for their studies.

**Table 1:** Study Subject Characteristics by Classification Group.

Group		Control (N = 36)	No Nodules (N = 35)	Solid Nodules (N = 55)	GGO (N = 46)	Cancer (N = 22)
AGE (years)	Median	55.5	56	59	56	64.5

Pack Years	Median	NA	43	39	45	52

Asbestos Yrs	Median	NA	25	18	12.5	0

FEV_1_/FVC	Median	NA	71	75	73.5	68.5

Years Follow Up	Median	NA	NA	3.0	2.5	NA

**Table 2:** Study Subject Medical Characteristics by Classification Group.

Group	Control # (%) 36(100%)	No Nodules # (%) 35(100%)	Solid Nodules # (%) 55(100%)	GGO # (%) 46(100%)	Cancer # (%) 22(100%)
Male	16(44.4%)	15(42.9%)	22(40%)	18(39.1%)	11(50%)

Female	20(55.6%)	20(57.1%)	33(60%)	28(60.9%)	11(50%)

Cancer on follow up	NA	0(0%)	0(0%)	5(10.9%)	0(0%)

SN or GGO Resolved	NA	0(0%)	5(9.1%)	6(13%)	0(0%)

Emphysema on CT	NA	11(31.4%)	17(30.9%)	21(45.7%)	9(40.9%)

Pleural Plaque(s)	NA	2(5.7%)	6(10.9%)	3(6.5%)	0(0%)

Fibrosis	NA	2(5.7%)	3(5.5%)	2(4.3%)	0(0%)

Diabetes	NA	3(8.6%)	8(14.5%)	5(10.9%)	2(9.1%)

Rheumatoid Arthritis	NA	0(0%)	2(3.6%)	1(2.2%)	1(4.5%)

Lupus	NA	0(0%)	0(0%)	0(0%)	1(4.5%)

IBD	NA	0(0%)	1(1.8%)	1(2.2%)	1(4.5%)

Psoriasis	NA	0(0%)	2(3.6%)	1(2.2%)	0(0%)

HIV	NA	0(0%)	0(0%)	1(2.2%)	0(0%)

Thyroid Disease	NA	2(5.7%)	8(14.5%)	6(13%)	3(13.6%)

Hepatitis	NA	1(2.9%)	2(3.6%)	4(8.7%)	1(4.5%)

Bronchiectasis	NA	0(0%)	4(7.3%)	3(6.5%)	1(4.5%)

Bronchiolitis	NA	0(0%)	2(3.6%)	3(6.5%)	0(0%)

Diffuse Nodular Disease	NA	0(0%)	2(3.6%)	2(4.3%)	0(0%)

Pneumonia	NA	0(0%)	0(0%)	3(6.5%)	0(0%)

Possible concurrent malignancy	NA	0(0%)	3(5.5%)	3(6.5%)	2(9.1%)

All participants in the NYU LCBC were administered a questionnaire including questions on medical history, occupational exposures, family history and respiratory symptoms. Spirometry was performed according to ATS criteria. Low dose chest CT using a multidetector scanner (16 detectors) was performed as described previously [3]. Follow-up scans were performed at defined intervals based on the initial CT scan findings. CT scans were reviewed by a radiologist and a pulmonologist. All study subjects signed an informed consent and the protocol was approved by the NYU School of Medicine Human Subjects Review Committee.

### Measurement of Immune Reactivity to TAAs

From previous studies [16,19,26], ten TAAs were selected to form a panel of TAAs for determining presence or absence of autoantibodies in each serum specimen. The TAAs consisted of purified full-length recombinant proteins: three insulin-like growth factor 2 mRNA binding proteins (IMP1, p62/IMP2 and koc/IMP3), p53, c-myc, cyclin A, cyclin B1, cyclin D1, CDK2 and survivin.

Purification of recombinant TAAs and enzyme-linked immunosorbent assay was performed as previously described using protein denaturing technologies [16,19,27]. Briefly, we subcloned p62 cDNA into the pET28a vector, producing a fusion protein with NH-terminal 6 × histidine and T7 epitope tags. The recombinant protein was affinity purified on nickel-nitrilotriacetic acid (Ni-NTA) columns according to the manufacturer's instructions (Qiagen, Inc., Valencia, CA, USA). IMP3/Koc cDNA cloned in the pcDNA3 vector was similarly subcloned into the pET28a vector and the recombinant protein was expressed as above. IMP1 construct, pCMV5-IMP1, was kindly provided by Dr F.C. Nielsen (University of Copenhagen, Copenhagen, Denmark), and p53 clone, p53SN3, by Dr Y. Yin (Columbia University, New York, NY). cDNA from c-myc was amplified by polymerase chain reaction from fetal liver tissue and survivin cDNA from human survivin EST clone (BG25843) before subcloning in the pET28a vector. Glutathione-S-transferase (GST)-cyclin D1 fusion protein was prepared from plasmid Gex (pGEX) containing cyclin D1 coding region obtained by amplification with polymerase chain reaction. CDK2 protein was from plasmid pRK171 containing PCR-amplified coding region of CDK2. CyclinB1 without a fusion protein partner was prepared from pRK171 expressing cyclin B1 and was a gift from C. McGowan (The Scripps Research Institute, La Jolla, CA) and pGEX expressing cyclin B1 with GST fusion partner was a gift from E. Harlow (Massachusetts General Hospital Cancer Center, Boston, MA). pRSET expressing cyclin A was a gift from T. Hunter (Salk Institute, La Jolla, CA) [16]. To produce recombinant p90, gel purified 1.2 kb EcoR1-XhoI insert from GC291 was excised from the pBK-CMV plasmid and subcloned into the pET28b expression vector (Novagen, Madison, WI) as previously described [28]. The pET construct was transformed into E. coli BL21 (DE3) (Stratagene, La Jolla, CA) for recombinant protein expression in the presence of 2 mM IPTG. After a four-hour incubation, recombinant proteins were extracted and purified using nickel-nitrilotriacetic acid (Ni-NTA) bead affinity columns (Qiagen, Valencia, VA) [25].

Enzyme-linked immunosorbent assay (ELISA) was performed as previously described [25]. Purified recombinant proteins were diluted in phosphate buffered saline (PBS) to a concentration of 0.5 micrograms/ml for coating wells of microtiter plates. Human sera diluted at 1:200 were incubated in the antigen-coated wells. Horseradish peroxidase-conjugated goat anti-human IgG (Caltag Laboratories, San Francisco, CA) was added and 2,2'-axinobis (3-ethylbenzthiazoline-6-sulfonic acid) (Boehringer Mannheim GmbH, Mannheim, Germany) was used as the substrate. Each sample was assessed in duplicate, and the average value at 490 nm was used for data analysis.

### Statistical Analysis

The objectives of this study were to distinguish lung cancer from other groups based on TAA levels, to compare the distributions of TAA levels among the groups, and to predict the presence of cancer based on these ten biomarkers.

	The distributions of demographic and medical history characteristics were summarized for the five groups of subjects under study using frequency distributions for categorical variables and summary statistics for continuous variables. For each group of samples, descriptive summary statistics (mean, median, and standard deviation) and box plots are provided for each of the 10 biomarkers under study.

Kruskal-Wallis nonparametric analysis of variance Chi Square tests were used to assess the differences in the distributions of individual biomarker measurements by classification group. These analyses were conducted to compare the four groups of lung cancer patients, GGOs, solid nodules, and nodules and for all five groups of subjects including the healthy controls. If the overall test statistic is significant (p ≤ 0.05, 2 -sided) for an individual biomarker, then pairwise Wilcoxon Rank Sum tests were used to identify the groups that are significantly different in their distributions of an individual TAA. A conservative Bonferroni adjustment for multiple testing was used for the analysis of each biomarker; no adjustment was used for the examination of the 10 biomarkers. Logistic regression models were developed to examine the prediction of group membership based on the individual biomarkers and combinations of biomarkers. Since the control group was from a different population (healthy, nonsmokers), we considered only the 158 subjects from the 4 groups in the NYU cohorts (lung cancers, GGO, solid nodule, and no nodules) in the analysis. Box Cox transformations were considered for the biomarker measurements and log transformations were selected for the regression analyses. Model fit was evaluated using the Akaike Information Criterion (AIC) with model selection based on minimizing the AIC. Two sets of multiple logistic regression models were considered. First, we used the logistic regression models to predict the presence or absence of cancer at the initial screening (combining the no nodules, solid nodules and ground glass opacity groups); second, we considered ground glass opacities vs. no ground glass opacities (combining the no nodules, solid nodules and cancer groups). The control group of healthy volunteers that was not included was used as a test set to evaluate the predictive ability of the models. Receiver Operating Characteristic (ROC) curves were created to plot sensitivity and specificity of the selected models for each outcome. All computations were performed using SPlus, SAS, and R.

## Results

The characteristics of the subjects enrolled in the five groups are shown in Table 1. We noted that the lung cancer patients were older (median age 64.5) compared with other subjects and had increased pack years of smoking (52) compared with other subjects; these lung cancer cases also had the lowest median FEV_1_/FVC (68.5). Gender and median pack-years smoking were comparable among the four smoking groups. The lung cancer patients tended to have slightly lower FEV_1_/FVC ratios compared with individuals without known cancer (lung cancer median 68.5% vs. smokers with normal CTs 71 ± 8%). Table 2 provides a summary of medical history for these groups of subjects. The nonsmoking controls had been screened to be in good health. Note that 5 (10.9%) subjects with ground glass opacities developed lung cancer on follow-up (13% resolved); 9.1% of patients with solid nodules also had resolution of these nodules on follow up (Table 2). Only the proportion of patients with emphysema and pneumonia differed among the groups with the highest proportions with these diseases on CT scan observed in the ground glass group (p ≤ 0.001, p = 0.04, respectively, chi square tests).

**Figure 1:**
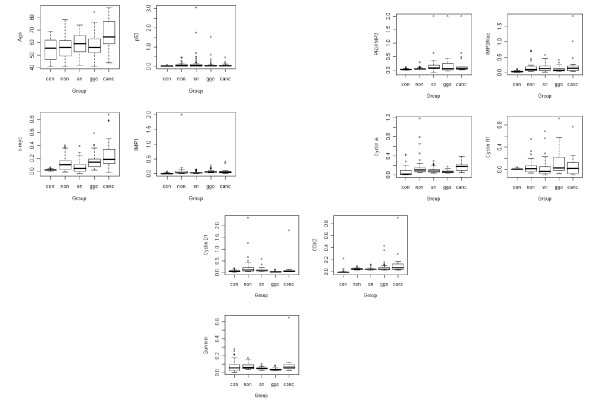
**Box plots of Age and Autoantibodies to TAAs by Classification Group**. TAAs: p53, c-myc, IMP1, P62/IMP2, IMP3/KOC, Cyclin A, Cyclin B1, Cyclin D1, CDK2, Survivin. Classification Group: con-control, non-no nodule, sn-solid nodule, ggo-ground glass opacities, canc-cancer.

All of the patients with lung cancer and most of the individuals with ground glass opacities demonstrated reactivity to one or more of the 10 TAAs, (using a cut-off value of > mean + 3 SD of non-smoking normal sera). Figure 1 provides box plots that summarize the distributions of age and of autoantibodies to each of the 10 TAAs for the five groups of subjects. The distributions of each of the 10 biomarkers differed among the five classification groups (p = 0.008 for cyclinB1; p < 0.001 for all others). After a conservative adjustment for multiple comparisons, we note that the healthy nonsmoking controls have significantly lower median levels of each of these biomarkers compared with the four groups of smokers including lung cancer cases. Table 3 summarizes the results of the pairwise Wilcoxon Rank Sum tests (bolded values are significant with a conservative Bonferroni adjustment for multiple testing) for the comparisons among the four groups of subjects in the NYU cohorts. In particular, the median levels of autoantibodies to Cyclin A, Cyclin D1, and survivin are lower in the group with ground glass opacities compared with the groups with cancer, no nodules, or solid nodules. The cancer group has significantly higher levels of autoantibodies to c-myc, Cyclin A, and CDK2 compared to the solid nodule group.

**Table 3:** Pairwise non-parametric Wilcoxon tests with Bonferroni adjustment to detect differences between classification groups and biomarker levels (comparisons to controls not shown)

		p53	c-myc	imp1	p62	imp3	cycA	cycB1	cycD1	cdk2	survivin
No Nodules	Solid Nodules	1.000	0.031	1.000	0.001	1.000	1.000	0.228	0.351	1.000	0.022

No Nodules	GGO	0.750	0.810	0.128	1.000	0.680	**<0.001**	1.000	**<0.001**	1.000	**<0.001**

No Nodules	Cancer	0.630	0.049	1.000	1.000	1.000	0.258	1.000	**0.001**	0.025	1.000

Solid Nodules	GGO	0.500	**<0.001**	**<0.001**	1.000	0.170	0.003	*0.007*	**<0.001**	0.423	**<0.001**

Solid Nodules	Cancer	0.630	**<0.001**	*0.009*	1.000	1.000	**<0.001**	1.000	0.018	0.001	0.139

GGO	Cancer	1.000	0.532	1.000	1.000	0.350	**<0.001**	1.000	**<0.001**	0.262	**<0.001**

**Figure 2:**
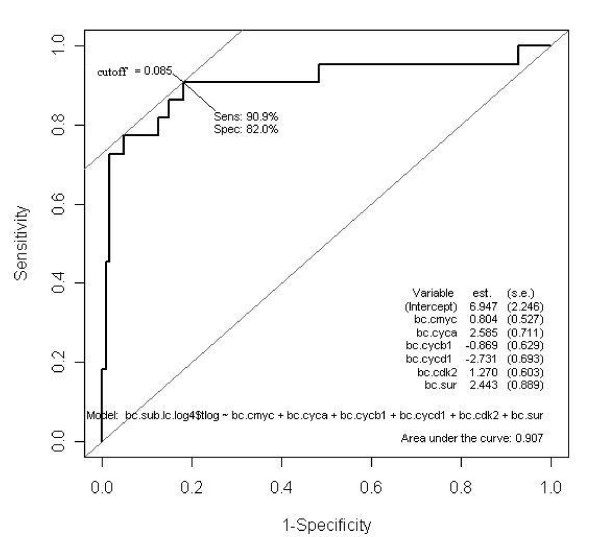
**ROC Curve Based on Stepwise Multiple Logistic Regression and Log Transformed Biomarkers to Classify Cancer/No Cancer (no nodules, solid nodules, and ground glass opacities groups)**.

One hundred forty nine of the 158 high-risk smokers and cancer cases had complete data on all 10 biomarkers. A multiple logistic model to predict cancer versus no cancer (no nodules, solid nodules, or ground glass opacities) yielded an AIC of 86.6 with 91% accuracy using 10 fold cross validation (Table 4A). The best stepwise model reduced the AIC to 79.4 with the same cross validation prediction accuracy (Table 4B). C-myc, cyclin A, cyclin B1, cyclin D1, CDK2, and survivin contributed to the separation between the cancer and non cancer groups. The resulting Receiver Operating Characteristic (ROC) curve is shown in Figure 2 along with the sensitivity and specificity associated with the optimal cutpoint (91%, 99% respectively). When this model, which includes c-myc, cyclin A, cyclin B1, cyclin D1, CDK2, and survivin, was applied to the healthy non-smoking control group as a test set, we correctly classified 31/36 control patients as non-cancer using a cutoff value of 0.085 which maximizes the sensitivity and specificity of the logistic function at 81% and 97% respectively. Removal of outliers yielded similar results.

**Table 4:** Multiple Logistic Regression Models for cancer vs no cancer (no nodules, solid nodules, and ground glass opacities groups) based on log transformed biomarkers on 149 subjects with complete data.

**A: All Biomarkers**.					
	
	Estimate	Std. Error	z value	Pr(>|z|)	
	
(Intercept)	6.39	2.52	2.54	0.01	
	
P53	-0.11	0.48	-0.22	0.82	
	
C-myc	0.93	0.56	1.66	0.10	
	
IMP1/Koc	-0.21	0.56	-0.37	0.71	
	
P62/IMP2	-0.30	0.49	-0.60	0.55	
	
IMP3	0.14	0.67	0.21	0.84	
	
Cyclin A	2.69	0.79	3.41	<0.01	
	
Cyclin B1	-0.84	0.69	-1.22	0.22	
	
Cyclin D1	-2.70	0.83	-3.27	<0.01	
	
CDK2	1.32	0.67	1.95	0.05	
	
Survivin	2.39	0.91	2.62	0.01	
	
AIC	86.60				
	
10 fold Cross Validation	91%				
	
**B: Stepwise Multiple Logistic Model**.					
	**Estimate**	**Odds Ratio**	**Std. Error**	**z value**	**Pr(>|z|)**

(Intercept)	6.95	1043.15	2.25	3.09	<0.01

C-myc	0.80	2.22	0.53	1.53	0.13

Cyclin A	2.59	13.32	0.71	3.64	<0.01

Cyclin B1	-0.87	0.41	0.63	-1.38	0.17

Cyclin D1	-2.73	0.06	0.69	-3.94	<0.01

CDK2	1.27	3.56	0.60	2.11	0.04

Survivin	2.44	11.47	0.89	2.75	0.01

AIC:	79.40				

10 fold Cross Validation	91%				

## Discussion and Conclusions

A biomarker for the early detection of lung cancer will likely require a panel of 4-10 markers to maximize sensitivity and specificity. High throughput technological advances allow for the rapid evaluation of a huge number of possible markers, for example, by microarray analysis of gene expression, or proteomic analysis using SELDI or MALDI technology. 2 D-PAGE has identified antioxidant enzymes, ATP synthases, β1,4-galatosyltransferase, glutathione-S-transferase, and ubiquitin thiolesterase among others to be increased up to 10-fold in lung cancer compared to uninvolved tissue and correlated with their gene expression [29]. Elevated levels of phosphoglycerate kinase 1 in the serum were significantly correlated with poor outcome in lung adenocarcinoma [30]. Protein expression profiling using MALDI-TOF of lung adenocarcinoma has identified unique peaks that were sequenced and found to match macrophage migration inhibitory factor and cyclophilin A that also were over-expressed in tumor tissue using immunohistochemistry [31]. In contrast, using tissue microarrays and immunohistochemistry for these two proteins in 234 lung cancer patients, Howard and colleagues found no correlation with outcome although both proteins were over-expressed in most NSCLC tumors [32]. We identified 7 of 84 antibodies using two-color rolling-circle amplification protein microarrays that gave a significant (p < 0.01) difference for 24 lung cancer patients compared to 24 controls and 32 COPD subjects [33]. Proteins identified were C-reactive protein, serum amyloid A, mucin 1, and α-1-antitrypsin. C- Reactive Protein and Serum Amyloid A may reflect the inflammatory milieu surrounding the lung tumor, or may reflect over-production of these as actual biomarkers. C-Reactive Protein along with lung function and pack-years of smoking predict progression of bronchial dysplastic lesions [34]. Recently 14-3-3 Theta and LAMR1 were added to Annexin I and PGP 9.5 achieving a ROC of 0.73 (0.69 to 0.81) with significant p values for three out of four autoantibodies comparing the samples drawn at 0-6 months to 7-12 months prior to diagnosis in 85 lung cancer and control paired samples from the Carotene and Retinol Efficacy Trial serum bank [35].

Zhong and colleagues used fluorescent microarray technology to identify immunogenic phage-expressed proteins from T7 phage NSCLC tumor libraries, and to assess the presence of corresponding antibodies in the plasma of patients with NSCLC [36,37]. Their five most predictive phage-expressed proteins, combined in a logistic regression analysis, achieved 90 percent sensitivity and 95 percent specificity in their population of advanced stage NSCLC. The leave-one-out statistical analysis achieved 88.9 percent diagnostic accuracy. A subsequent study using similar antibody profiling in patients with early stage lung cancer from a CT screening study identified a slightly different panel of five antibody markers that was able to correctly classify stage I NSCLC with 91 percent accuracy, and predicted the presence of lung cancer up to five years before diagnosis with a sensitivity of almost 83 percent [18]. However, this panel of markers did not perform as well in identifying bronchoalveolar cell carcinoma. Proteins of interest were the heat shock proteins, proto-oncogenes, ras-associated oncogene, transcriptional regulators, DNA mismatch repair, tumor necrosis factor receptor, paxillin (focal adhesion protein), and several unknown proteins. Plakophilin 3 and ubiquilin 1 have been found to be humoral response targets using lung cancer phage display libraries [38,39]. Serum tumor markers such as carcinoembryonic antigen have been reported to be positive in 46 percent of 200 lung adenocarcinoma patients, as well as CA-125 and cytokeratin 19, but these markers correlate better with tumor burden or advanced stage [40].

We have taken a different approach, and focused on known proteins that play a role in the development of lung cancer. We included three proteins containing similar RNA-binding motifs, that we and others have shown to be over-expressed in cancers and where antibodies to these TAAs have been identified in cancer patients: IMP1 (insulin-like growth factor 2 mRNA binding protein), IMP2/p62, and IMP3/Koc [12,19]. We also included the tumor suppressor gene product p53 and oncogene c-myc, proteins known to be aberrantly expressed in early lung cancers. Survivin is an anti-apoptotic protein that we have shown to be antigenic in cancer patients [23]. The cell cycle proteins cyclin A, cyclin B1 (shown to be antigenic in several malignancies), cyclin D1 and CDK2 were also included. Zhang and colleagues studied sera of 777 cancer patients with 20/84 (24 percent) of lung cancer patients having one or more autoantibodies to this panel of antigens [19].

The objective of this study was to distinguish biomarker measurements among five classifications based on the CT scan: Non-smoking controls, no nodules, solid nodules, ground glass opacities, and cancer. Based on the nonsmoking and healthy smoker controls, patients with lung cancer had increased levels of immune reactivity to the TAAs. Smokers with benign solid nodules and smokers with no nodules had equivalent lower levels of reactivity. Smokers with GGOs, which may represent a preneoplastic or neoplastic condition in some patients, had an intermediate level of reactivity between patients with cancer and smokers with normal CTs or benign solid nodules. We found that reactivity to two tumor associated antigens, c-myc and p62, correlated most closely with the presence of either GGOs or lung cancer. From these analyses, it appears that it is possible to distinguish cancer and ground glass opacity patients based on their biomarker measurements with some measure of certainty. Cyclin A, Cyclin D1, CDK2, and survivin appear most often in models, irrespective of outcome. However, this study is limited by small sample size, of which only 22 patients had cancer and extreme biomarker measurements could not be repeated or validated.

Persistent GGOs are more likely to represent preneoplastic or neoplastic lesions. Nakata and colleagues reported that 100 percent of ground glass opacities ≤ 2 cm in size that persisted for at least three months were found to be malignant or pre-malignant lesions on biopsy or resection [41]. Many persistent GGOs are likely to be adenocarcinoma, bronchoalveolar carcinoma, or atypical adenomatous hyperplasia, and assessing these versus benign nodules or smoker controls using autoantibodies to TAAs is unique. Combinations of antibodies against TAAs are most likely to achieve the necessary sensitivity and specificity for early detection in CT scan screening trials when small noncalcified solid or ground glass nodules are discovered in the >8 mm size range. These prediction models require validation in larger patient groups with samples collected prospectively.

## Competing interests

The authors declare that they have no competing interests.

## Authors' contributions

WR conceived and organized the project and drafted the manuscript. JG and HW performed biostatistical analyses, DAH, MK, AG, and EE evaluated the high-risk smokers and AG assisted in drafting the manuscript; DN provided the radiology readings of the CT-scans; BC examined the lung cancer patients, and ET and DL performed the autoantibody assays. All authors read and approved the final manuscript.

## Pre-publication history

The pre-publication history for this paper can be accessed here:

http://www.biomedcentral.com/1471-2407/10/234/prepub
